# Soundscape experience activities and mapping

**DOI:** 10.1038/s44384-025-00041-6

**Published:** 2026-02-23

**Authors:** Thomas Deacon, David Frohlich, Mark D. Plumbley

**Affiliations:** 1 Centre for Vision, Speech and Signal Processing, University of Surrey, Guildford, UK; 2 Digital World Research Centre, School of Arts, Humanities and Creative Industries, University of Surrey, Guildford, UK

**Keywords:** Neuroscience, Psychology, Psychology

## Abstract

This paper presents Soundscape Experience Activities and Mapping (SEAM), a new method for exploring how older adults perceive and relate to their indoor acoustic environments. With global ageing, populations are increasingly choosing to age in place, creating opportunities to enhance older life through the intentional design of supportive home soundscapes. Through a mixed-method approach combining Ecological Momentary Assessment with Cultural Probe methods, we engaged eight older adults (age 56–76) in Belgium to document their domestic soundscape experiences. Reflexive thematic analysis constructed four patterns of meaning: personal agency in shaping acoustic environments, temporal routines structured by sound, sound-memory associations fostering place attachment, and social presence through acoustic monitoring. Within this study context, sounds functioned as spatiotemporal anchors, structuring daily routines while fostering place attachment through memory. This exploratory design research offers situated insights for soundscape interventions that support independence, while highlighting methodological considerations for situated soundscape research.

## Introduction

As the global population aged 60+ is projected to grow by roughly a third between 2019 and 2030^1^, ageing-in-place has become a dominant policy and life course^2^. Supportive homes for ageing-in-place work by increasing residents’ ability to shape conditions for sound, light and thermal comfort, and by preserving agency and routine^3^. Poor acoustic conditions, such as everyday exposure to intrusive noise, can accumulate into chronic stress and disturbed rest, with cardiovascular implications^4^, as well as impacting a person’s sense of control over their sonic environment^5^. So, creating supportive indoor sound environments for older adults is an important facet of public health design^6^.

Yet older adults face a distinct challenge: hearing changes with age. A systematic review of soundscape perception shows older adults differ from younger groups across noise annoyance, acoustic comfort, and sound preference^7^. Age-related hearing changes alter how sounds are heard, segregated and appraised^8–10^. An older adult may struggle to follow a conversation when a ventilation fan hums in the background, or find that street noise disrupts their afternoon rest. Furthermore, noise may also influence late-life cognition and risk of dementia^11^.

Indoor soundscape research supports tackling this challenge by framing acoustic experience as perceiver-centred^5^. Building on soundscape research, where human experience of the acoustic environment is shaped by context, activity, expectation and meaning, not just decibels^5,12^. Indoor soundscapes differ from outdoor ones, offering greater potential for control and predictability yet being shaped by building systems and occupant activities^5^.

This potential to create supportive acoustic spaces through sonic control is where we began exploring how innovations in Audio AI could help. The AI for Sound (AI4S) project (http://ai4s.surrey.ac.uk) explores how audio machine learning might address societal challenges. Audio AI encompasses machine listening and generative audio technologies, which offer new ways to analyse^13,14^ and shape acoustic environments^15^. For the home, our goal became to enhance well-being by minimising unwanted sounds, amplifying desired ones, and offering personal control. But, given limited data or methods on older adults’ home soundscapes, we looked to in situ methods to gather data and derive requirements.

Indoor soundscape assessment builds on established perceptual attributes (pleasant, vibrant, eventful, chaotic, annoying, monotonous, uneventful, calm) while adding indoor-specific dimensions such as ‘private/under-control’ versus ‘intrusive/uncontrolled’^16^. Perception varies with location, activity, and social context^17^, and recent work is working these situational factors into ISO frameworks^18^. For older adults at home, research suggests such factors may be particularly salient given distinct perceptual needs^19^. In parallel, developments in *affective listening* emphasise how sounds elicit felt, embodied, and relational responses in context^20,21^, reinforcing a multi-faceted view of indoor soundscape perception.

Sense of place (SoP) offers a complementary lens on how environments become meaningful^22^, integrating sensory and cognitive dimensions that include indoor soundscapes. It comprises place attachment (emotional bonds) and place identity (self-relevant meanings)^22^. In the home, soundscapes can act as emotional anchors that help build residents’ SoP^23,24^; this role came into sharp relief during COVID-19 lockdowns, when domestic acoustic environments mediated comfort, content, privacy, and routine under work-from-home conditions^25,26^.

Sound experience also engages multiple memory systems^27^. From sensory processing to long-term storage via episodic memory (personal experiences) and semantic memory (sound knowledge), these systems help familiar soundscapes confer personal significance on domestic spaces^28,29^. Expectations formed in the home shape subsequent perception^28^, with both acoustical properties and psychological associations influencing appraisal^30^.

Complementary methods for capturing situational factors and sense of place are considered effective for studying indoor soundscapes^6^. Two strands recur in work on homes, sound, and technology: in situ sampling of soundscape appraisals, and design-led elicitation that surfaces tacit experience. Ecological Momentary Assessment (EMA) captures in situ appraisal, affect, and situational details^31^, enabling analyses of how perceived soundscape attributes vary with activities and settings^17^. Large-scale applications in homes indicate that perceived loudness and appraisal are only partly explained by level, with situational and perceptual attributes adding explanatory value^32^. Design research methods such as guided sound tours^33^ and Cultural Probes^34^ (applied in “Cultural probe activities and soundscape notepad” section), prompt reflection and self-documentation, revealing overlooked sounds, language, and constraints; they surface home-specific concerns and design opportunities. This is achieved by active engagement with sound through novel activities such as sound journalling and recording^35^. More broadly, Human-Computer Interaction (HCI) studies of the home emphasise sensitivity to established routines and communication ecologies^36^, to ensure new innovations do not disrupt the “stable routines of the home”^37^.

Our purpose in this research is to explore how older adults in our study context construct meaning from their home soundscapes, generating situated insights to inform later design and engineering of supportive indoor soundscape systems. To do this, we developed Soundscape Experience Activities and Mapping (SEAM), a participatory approach centred on lived experience. This study pursues three aims:

1. **Methods:** develop a participatory methodology for capturing domestic soundscape needs in context;
2. **Experience:** identify situational factors shaping perception and appraisal of home soundscapes; and
3. **Positioning:** articulate design directions for future indoor soundscape research grounded in these experiences.

This paper reports a novel methodology for collecting domestic soundscape experiences, findings from listening tasks, and participant feedback on home soundscape studies with older adults. Audio AI applications are briefly discussed in future work addressing the design translation of qualitative findings. As a situated design research method, SEAM clarifies needs and constraints for later design work.

## Methods

Our aim was to characterise older adult participants’ everyday home soundscapes and how these are experienced. We report findings from an exploratory phase of a larger co-design project to build Audio AI systems for indoor soundscapes, focusing on in-home engagement and post-deployment reflections.

### Study design and participant recruitment

The study was delivered with Living and Care Lab (LiCalab, Geel, Belgium), which supported recruitment from their user panel, access to homes, and translation. Fieldwork took place in participants’ homes in the Geel municipality and at LiCalab facilities in Turnhout. The work was supported by the VITALISE programme; ethics approvals were obtained from the relevant boards.

**Fig. 1:**
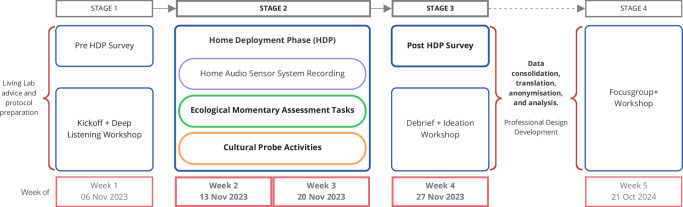
Four-stage living lab study timeline. Study conducted between November 2023 and October 2024. Areas covered in this paper’s results are highlighted with bold type.

Figure 1 summarises the four engagement stages. This paper reports Stage 2 (Home Deployment Phase with EMA and Cultural Probe tasks) and Stage 3 (post-deployment survey). Beyond the listening-focused elements reported here, the wider study included workshops, home audio recording of the living room and kitchen for 7 days, and an end-cap co-design workshop on Audio AI system proposals. These elements will impact the study experience of participants but are out of scope for the present analysis; see “Data availability” for details.

LiCalab recruited eight older adults (ages 56–76; 6 female, 2 male) from its user panel to meet inclusion criteria (healthy adults, able to use a smartphone), via convenience and snowball sampling. Most had prior research involvement with LiCalab. All participants were recruited from the Geel municipality in Flanders, Belgium, where our co-design work was situated. Table 1 summarises living arrangements. The panel manager noted that the requirement to have home audio recording devices installed for 7 days did make recruitment difficult, compared to other types of studies.

**Table 1:** Participant living arrangements and household composition

PID	Age	Sex	Living context	Co-living context	YIR
P1	56	F	Urban one-storey flat, no garden	Lives alone, no pets	5
P2	71	F	Suburban bungalow with garden	Lives with partner, no pets	46
P3	74	F	Rural bungalow with garden	Lives alone, no pets	6
P4	76	M	Suburban house with garden	Lives with partner*, no pets	38
P5	73	F	Suburban house with garden	Lives with partner*, no pets	38
P6	63	M	Rural house with garden	Lives alone in one wing of house, cares for elderly parent located in other wing of home, with pet (cat)	30
P7	65	F	Rural house with garden	Lives with partner, no pets	28
P8	69	F	Urban two-storey flat, no garden	Lives alone, with pets (cat, dog)	14

*PID* participant ID, *YIR* years in residence of current home.

*P4 and P5 are a couple.

Participants varied in age by 20 years, with living arrangements ranging from independent urban flats to rural homes with family care responsibilities, and with a mix of prior experience with research and technology. This heterogeneity was appropriate for our interpretive aims: to explore how soundscape meanings are constructed across diverse older adult circumstances rather than to establish statistical generalisations^38^. Comparable small-N, interpretive studies have applied cultural probes (10 families^35^), audio self-recording (7 households^33^), and situated observation (one family^39^) to understand lived experience in domestic and elder-care contexts.

### Ethics and informed consent

Participants physically signed informed consent for the study and data analysis with support from the LiCalab panel manager, who explained the protocol and addressed queries during the Stage 1 kickoff workshop. LiCalab maintained paper records to preserve participant anonymity, with authors using only numerical participant IDs throughout the data collection and documentation process. The study received ethical approval from all required boards (University of Surrey RIGO: FEPS 23-24 001 EGA and KU Leuven SMEC: G-2023 11 2174). All informed consent statements and information available in the workflow supplement (see “Data availability”).

### Soundscape experience activities and mapping approach

The Soundscape Experience Activities and Mapping (SEAM) combines activities designed to elicit creative interpretations of sound experience with the participatory capture of everyday indoor soundscapes. In-home tasks prompt both structured and free-form descriptions. As a research process, we characterise our method as *crafting invitations to listen*, engaging both our participants and ourselves as researchers in a reflexive dialogue.

**Fig. 2:**
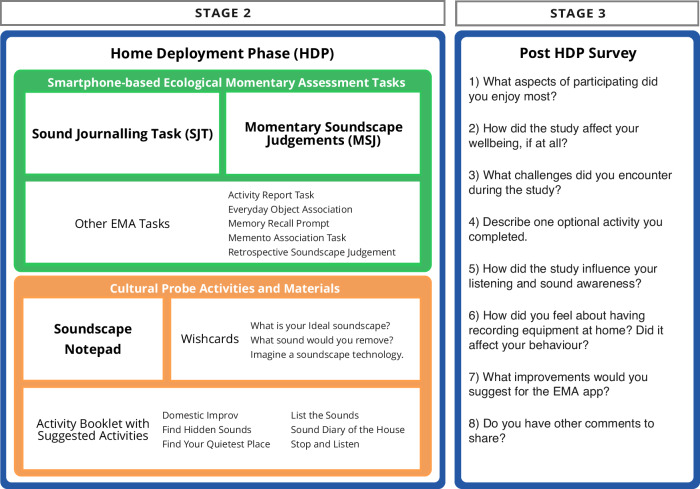
Home deployment phase data collection methods and instruments. The figure distinguishes smartphone-based ecological momentary assessment (EMA) tasks from physical cultural probe activities during Stage 2 (home deployment phase). Smartphone tasks included timed prompts for soundscape ratings, audio recordings, and contextual annotations. Cultural probe activities comprised diary entries, sketching exercises, and photographic documentation of meaningful acoustic environments. Post home deployment phase survey items (Stage 3) are shown in the right-hand panel. Bold typeface indicates data collection instruments reported in the “Results” section of this paper.

We combined workshop methods^40^, sound categorisation^41^, deep listening practices^21^, smartphone EMA^17,31^, and Cultural Probes^34^ to engage with situated soundscape experience. The SEAM protocol collected mixed-methods listening data through two complementary routes during the HDP (see Fig. 2): smartphone-based EMA tasks (“Ecological momentary assessment soundscape tasks” section) and Cultural Probe activities (“Cultural probe activities and soundscape notepad” section). A configurable smartphone EMA app (Avicenna Research^42^) enabled brief recordings and journalling, with notifications during deployment. The EMA app delivered structured tasks, including Momentary Soundscape Judgements (MSJ) and Sound Journaling Tasks (SJT). Additionally, the Cultural Probe provided materials for reflective exercises, including a *Soundscape Notepad* and optional Deep Listening Activities.

### Ecological momentary assessment soundscape tasks

The MSJ and SJT EMA listening tasks components in Table 2 summarise task steps, the experiential focus probed, and the elicitation approach. Other EMA tasks are documented in the “Data availability” section. The MSJ and SJT protocol was developed by integrating ISO 12913-2:2018 soundscape assessment data standards^43^ with EMA approaches^17,31,32^. Both MSJ and SJT tasks were delivered through smartphone notifications via the EMA app. MSJ tasks were prompted by daily notification, alternating between AM and PM periods, and were also available as a self-report option. The MSJ task was designed to take no longer than 5 min. SJT tasks were prompted by notification three times during the study week (on Days 2, 4, and 6 at specific times) and were also available as a self-report option. The task itself was described as taking around 15 min to complete, though the audio recording part was recommended to be under 5 min. Following other EMA Soundscape methods^17^, both the MSJ and SJT tasks asked participants to report on their current situational factors, including physical context, social context, and immediate emotional state^44^. Situational factors prompts and response categories are listed in the “Data availability” section, but are not required for the analyses in this paper.

**Table 2:** Ecological momentary assessment listening tasks used in this study

Task	Experiential focus	Elicitation approach
MSJ	Listening and recording	Participant records a 10–30 s environmental audio sample.
MSJ	Perceptual appraisal	Semantic-differential ratings on soundscape attributes (Pleasant-Annoying, Calm-Chaotic, Vibrant-Monotonous, Eventful-Uneventful; Private/Controlled-Intrusive/Uncontrolled).
MSJ	Sound tagging	Free-text listing of identifiable sound sources and qualities to capture descriptive categories.
MSJ	Affective reflection	Immediate thoughts and feelings; free text.
MSJ	Situational factors	See “Data availability”
SJT	Listening and recording	Participant captures a salient sound using video, audio+photo, audio+text, or text-only.
SJT	Meaning-making	Narrative on significance, context, and memory of the captured sound.
SJT	Affective reflection	Reflective account of why the sound mattered and how it felt; text or voice recording
SJT	Situational factors	See “Data availability”

Full item lists, response types, and scale visuals are provided in the “Data availability” section.

*MSJ* momentary soundscape judgement, *SJT* sound journalling task.

#### Momentary soundscape judgements

The assessment approach combined semantic-differential scales with free-form descriptions to balance standardised measurement with participant flexibility, following recommendations for soundscape assessment^41^. Participants listened and recorded a 10–30 s environmental audio sample, then rated their perception on indoor soundscape semantic-differential scales and answered two prompts:

1. *Try to identify all the different sounds you could hear, describe these in whatever way you like*. (Example response: “Nature, birds, waking up…cleaning up start the day” P6)
2. *What is going through your mind? Describe your thoughts and feelings after listening to the environment*. (Example response: “This is the atmosphere she should feel every day, the power of nature, letting things happen.” P6)

Belgium has three official languages: Dutch-Flemish in Flanders, French in Wallonia, and German in a small eastern region. The study was situated in Flanders, so we used validated Dutch-Flemish translations of soundscape attributes^45^ (see Table 3 for full details). The full MSJ item list is provided in the “Data availability” section.

**Table 3:** Dutch-Flemish soundscape descriptors used in the momentary soundscape judgement task

*English*	*Used wording (Dutch-Flemish)*
Pleasant	Aangenaam/Prettig
Annoying	Onaangenaam/Onprettig
Calm	Kalm/Rustgevend
Chaotic	Chaotisch/Hectisch
Vibrant	Levendig/Vrolijk
Monotonous	Saai/Eentonig
Eventful	Druk/Dynamisch
Uneventful	Rustig/Statisch

#### Sound journaling tasks

In the SJT, participants captured a salient sound and then provided a reflective narrative on its significance. Participants were prompted to “close your eyes and listen” and reflect on sound familiarity, documenting sounds through video, audio with photo, audio with text, or text alone. Recordings were limited to 5 min, with guidance to frame sound sources when possible. After capturing sounds, participants reflected on personal significance and emotional resonance. Example response from P4: “When we were walking this morning, it was wonderful to walk through a pack of large leaves. This sound is so specific that I have walked on that pile several times. The sound I heard is between crispy and fluffy, depending on the moisture of the pack of leaves. Really the time of my childhood with the difference that I did not pay so much attention to the sound then and now as a 76 year old.” Detailed SJT item flow and prompts are provided in the “Data availability” section.

Both the MSJ and SJT tasks were designed to capture participants’ experiences of sound, but they engaged different modes of sensory attention and reflection. The MSJ focused on immediate evaluation via standardised scales and brief reflection. The SJT supported deliberate, selective listening and deeper reflection on personally meaningful sounds. This complementary approach allowed us to capture both the breadth of ambient soundscape experience (through MSJ) and the depth of specific sound encounters (through SJT).

### Cultural probe activities and soundscape notepad

**Fig. 3:**
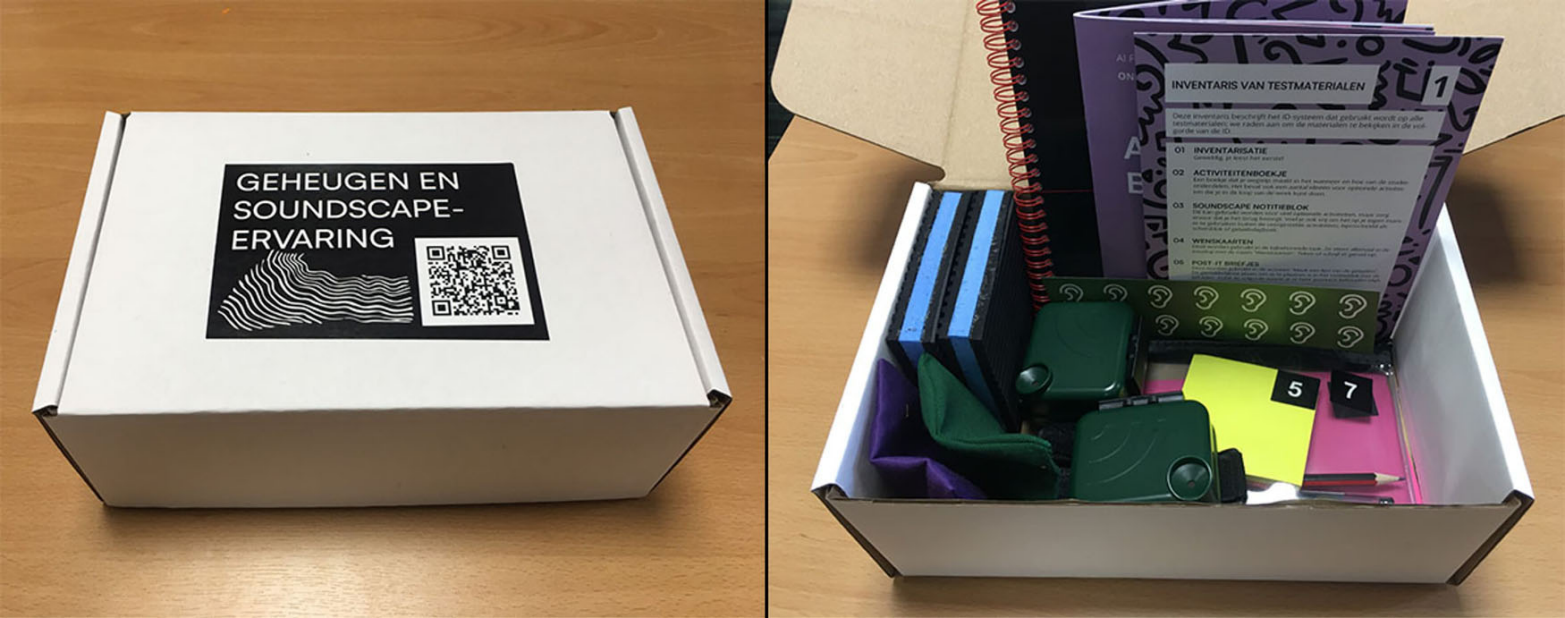
Cultural probe kit for soundscape experience activities and mapping study. The kit contains materials designed to facilitate participant documentation of domestic soundscape experiences. Image shows the closed probe box with Dutch title “GEHEUGEN EN SOUNDSCAPE-ERVARING” (Memory and Soundscape Experience); and the contents of the probe kit, including instructional materials, audio recording devices, notebooks, and colour-coded activity cards.

To complement our digital EMA tasks, we used Cultural Probe methods; participant-led research tools that offer materials and activities designed to gather rich, reflective data about subjective experiences^34,35,46^. The Cultural Probe kit can be seen in Fig. 3.

**Fig. 4:**
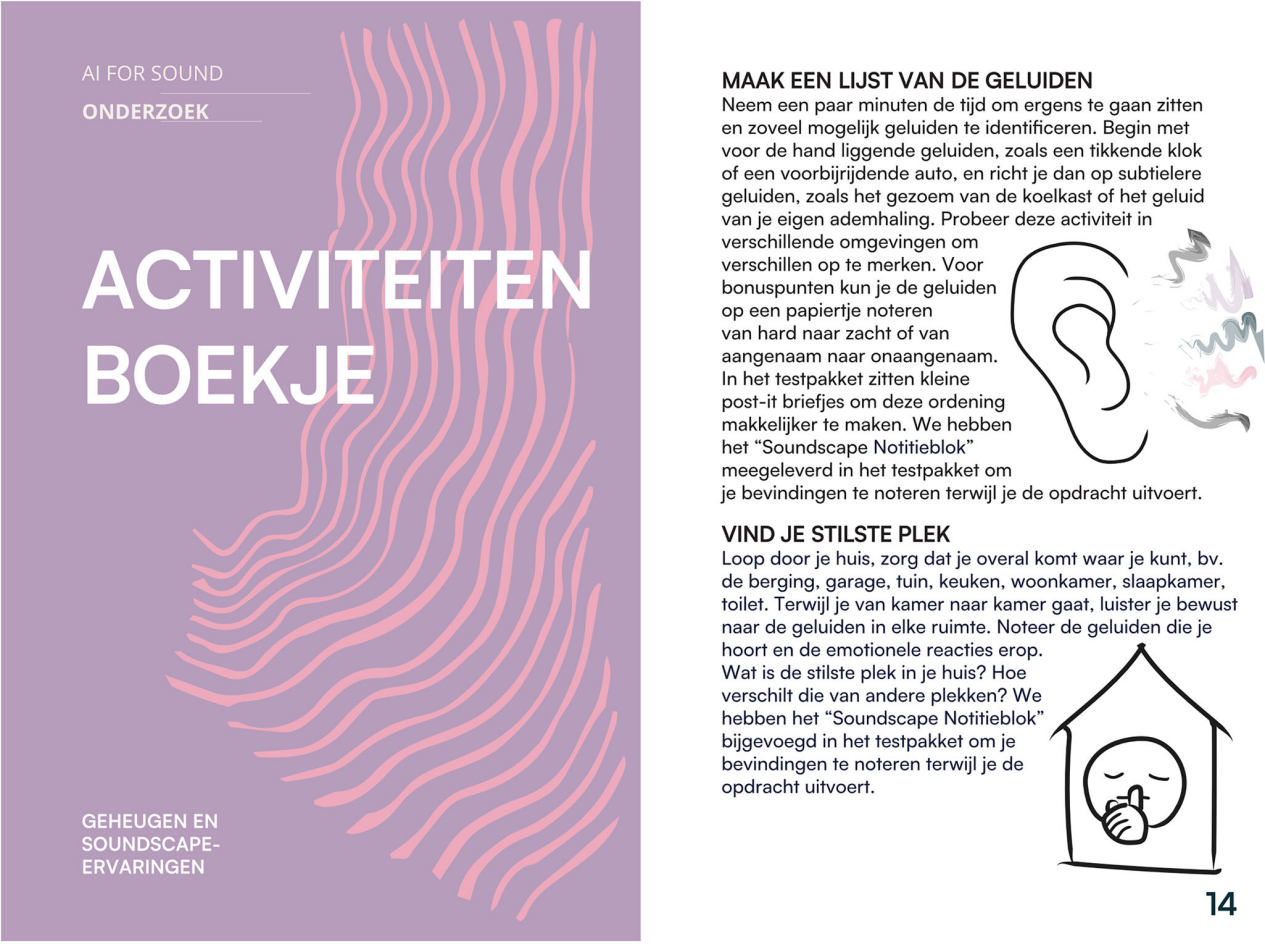
Activity booklet and sample activities. The probe kit contained materials designed to facilitate participant documentation of domestic soundscape experiences. Image shows Activity Booklet cover and sample page showing instructions for sound listening exercises, such as “Find Your Quietest Place” (VIND JE STILSTE PLEK).

**Fig. 5:**
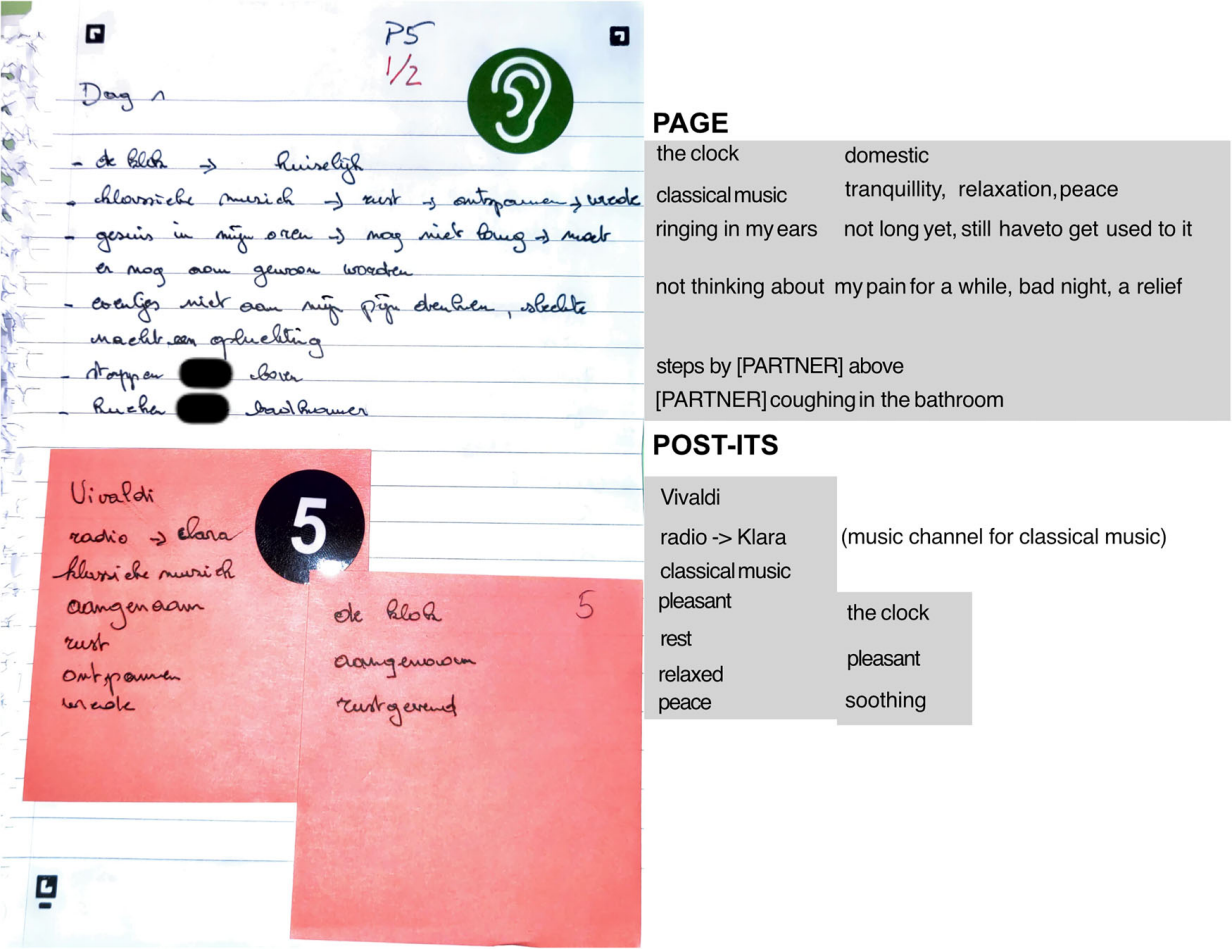
Soundscape notebook translation sample. Sample of a completed soundscape notebook entry showing participant documentation of sounds, their qualities, and associated emotions. The right-hand section shows how translation kept the spatial information of the original participant account.

The physical components included an Activity Booklet (Fig. 4) that guided participants through various listening exercises and reflective tasks. This booklet contained three main sections: (1) probe component overviews and usage tips, (2) deep listening guidance, and (3) suggested Deep Listening Activities to enhance sound awareness. Table 4 summarises each activity and its aim; participant copy of activities is available in the “Data availability” section. The kit supported a flexible set of activities—for example, “List the Sounds”, “Find Your Quietest Place”, and “Sound Diary of the House”—designed to build personal listening practice and sound awareness. Activities were categorised using simple icons to indicate their focus (deep listening, memory, domestic, or non-domestic), allowing participants to select exercises matching their interests and comfort levels. Additionally, the probe kit included a *Soundscape Notepad* (Fig. 5), a paper notebook for noting observations during listening activities.

**Table 4:** Cultural probe activities and aims

Activity	Aim
List the sounds	Increase auditory attention by enumerating salient and subtle sounds across contexts.
Find your quietest place	Map acoustic variation at home and reflect on the qualities of perceived quiet.
Sound diary of the house	Observe daily temporal patterns in domestic sounds and associated feelings.
Wish cards	Articulate desired changes to everyday soundscapes and speculative design ideas.
Find hidden sounds	Notice sources that are heard but not seen to expand listening beyond vision.
Domestic improvisation	Explore sound-making affordances of household objects through playful experimentation.
Stop and listen	Practice brief, eyes-closed situational listening to surface reactions and appraisals.

### Reflexive thematic analysis

This study employed Reflexive Thematic Analysis (RTA)^47,48^ to analyse qualitative data from participant responses and discussions. The analysis focused on identifying, coding, and interpreting patterns of meaning within the data^47^, with particular attention to participants’ lived experiences.

**Fig. 6:**
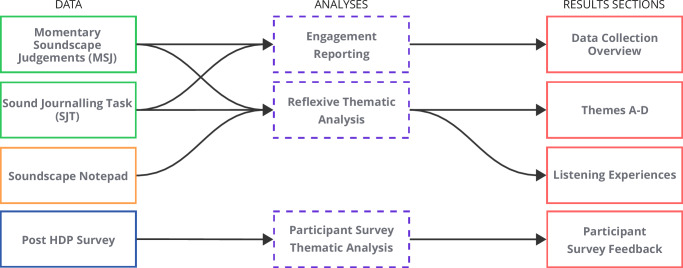
Data-analysis-results mapping. Shows the relationship of method components to analytical processes and paper sections.

The analytical process followed five steps (full analytical process available on request):

1. Collection and grouping of similar observations (e.g., annoyance of machine sounds);
2. Development of logical explanations for observed patterns (e.g., sounds are disruptive to the sense of tranquillity);
3. Identification of potential mechanisms and relevant theory supporting these patterns (e.g., patterns of sound and memory of annoying sounds, spatial cognition, social interpretation of the cause of annoying sound);
4. Reporting of themes and *Sensitising Concepts* (see below);

The analysis was conducted using MAXQDA24 software^49^, and the findings were further evaluated through project team data workshops to refine the interpretations and design implications. The flow of sources to findings, via analysis methods, is shown in Fig. 6.

Themes are based on their conceptual significance rather than statistical prevalence, drawing on Sensitising Concepts (SC) as orienting constructs rather than fixed categories^38,50^. Following Reflexive Thematic Analysis principles, themes are actively constructed through interpretive engagement with data. They are not ‘discovered’ or ‘emerge’ from it, as Braun and Clarke emphasise, “themes don’t emerge”, they are created by researchers^48^. This inductive approach prioritises meaning over frequency: a theme’s significance lies in its capacity to capture important aspects of the research question, even when evident in a few participant responses. This contrasts with coding reliability approaches that equate theme importance with frequency of occurrence^48^. Our approach aligns with guidance on evaluating qualitative research in HCI^38^, where interpretive validity rests on the apodicity (self-evidence) of findings as demonstrated through data rather than sample size or demographic representativeness. This interpretive stance does not preclude attention to variation: where relevant, we note how themes were expressed differently across participants’ varied circumstances (e.g., differences in living arrangements).

### Positionality

As a team (T.D., D.F., M.P.) with similar demographic attributes (white, male, over 40) working in a single Global North institution, we recognise that our perspectives reflect cultural and social contexts that shape how we frame questions, make methodological choices, and interpret data. Our experiences of sound environments differ from those of our participants, and our interpretations may inadvertently centre normative assumptions about listening, technology use, social relations, and the home. We addressed this through reflexive practice, transparent documentation, and participant-centred approaches. We prioritised participant language in analysis and adopted a stance of “hearing with” rather than merely “listening to” participants, and created space for diverse sonic experiences. Still, our positionality shapes the knowledge produced, and alternative interpretations would likely emerge from differently positioned teams. We encourage readers to critically engage with our findings and consider how different perspectives might yield alternative understandings of the phenomena we study, supporting diversity in soundscape research^51^.

## Results

### Data collection overview

The study collected 76 responses across MSJ (*N* = 38) and SJT (*N* = 38) tasks. Participants were requested to complete 7 MSJ and 3 SJT entries during the study period, achieving an average compliance rate of 68% (*σ*: 31%). Compliance varied across participants: some (P3, P4, P5, P6) completed all requested entries, while P3 contributed 20 SJT entries and P6 completed 9 MSJ entries. Full participant-level response rates are in the Supplementary Information (Note 1).

### Thematic analysis of sound experiences in the home

The thematic analysis of SJT and MSJ responses was driven by the guiding research question: *How did the older adults in our study perceive and make meaning from their domestic soundscapes in everyday life?* In the presentation of participant data, we use the coding system MSJ/SJT-XXPY, where MSJ/SJT is the task, XX is a unique data identifier for that task, and PY is the participant ID. Sensitising concepts are denoted as ‘SC’. We present four themes (A–D) addressing participants’ lived soundscape experience:

- **Theme A: Personal Agency and Environmental Response**
  - SC A1: Direct control and management of sound “I chose this myself”
  - SC A2: Subjective comfort experiences “the sound of the wind”
- **Theme B: The Steady Rhythm of Home Soundscapes**
  - SC B1: Practical structure “day-to-day sound that you hear”
  - SC B2: Ritual and comfort “comforting process”
  - SC B3: Spatiotemporal integration “evening takes over quietly”
- **Theme C: Sound and Memory Associations**
  - SC C1: Sonic place-making “peace, warmth, and home-like comfort”
  - SC C2: Memory bridge “brings me back”
- **Theme D: Social and Cultural Dimensions**
  - SC D1: Monitoring others “some chatter to hear at a distance”
  - SC D2: Cultural connections “geese high in the air”

The four themes represent our interpretation of patterns across accounts utilising the framing of sensitising concepts. We present them as design-relevant insights grounded in this specific study context rather than generalisable findings about all older adults.

#### Theme A: Personal agency and environmental response

Participants’ accounts suggest an interplay between personal agency and environmental response in their domestic soundscapes. Participants actively shaped their sonic environments through deliberate choices while passively encountering uncontrollable sounds, creating a dynamic between controlled and uncontrolled acoustic experiences.

##### SC A1: Direct control and management of sound “I chose this myself”

Participants demonstrated personal control through three behaviours. First, deliberate sound selection for comfort: “I feel comfortable because I chose this myself” (SJT-23-P3). Second, active sound management, such as one participant repeatedly walking through leaves to experience the sound (SJT-24-P4). Third, curated musical experiences, like selecting classical music for emotional comfort (SJT-32-P3).

Conversely, participants expressed frustration with uncontrollable sounds, particularly industrial noise and traffic. Four responses (SJT-37-P6, MSJ-03-P6, MSJ-11-P6, MSJ-28-P4) indicated feelings of helplessness regarding intrusive sounds, with this inability to control distressing sounds appearing in 11 responses across 3 participants.

##### SC A2: Subjective comfort experiences “the sound of the wind”

Participants described varied reactions to indoor soundscapes. We interpreted recurring associations between pleasant experiences and natural elements, and between disruption and industrial noise. However, individual context and preference often counteracted our expectations (note the high level of P3 responses).

Natural elements like “the sound of the wind” (MSJ-19-P3) or crunching leaves (SJT-24-P4) were associated with peace. Music created positive emotional experiences (SJT-02-P3, MSJ-23-P5, SJT-32-P3, MSJ-15-P5); consistent with older adult soundscape research^19^. Mechanical sounds were often invasive, with one participant noting people “have to go to a retreat or a forest to experience silence” (MSJ-11-P6). Participants expressed concern about noise pollution (MSJ-03-P6, MSJ-07-P6, MSJ-08-P3, MSJ-28-P4) with planes and traffic disrupting peace, e.g., “Noise outside a mowing machine … I live in a very quiet place and every sound that comes in I hear extra” (SJT-14-P3).

However, everyday domestic sounds could contribute to comfort depending on context. The same sounds could be either comforting or disturbing—a fridge sound was either soothing, “the soft buzzing of the fridge. A home-like feeling of peace” (MSJ-16-P6), or irritating, “buzzing of the fridge, monotonous and sharp, annoying” (MSJ-11-P6) for P6. This highlights nuanced contextual preferences for indoor soundscapes. The diversity of responses within individual participants (e.g., P6’s contrasting fridge experiences) and across the age range represented (from P6, aged 63, to P4, aged 76) highlights how contextual factors and momentary circumstances shaped soundscape perception.

#### Theme B: The steady rhythm of home soundscapes

Participants described domestic soundscapes as contributing to temporal stability and psychological security, with sounds serving multiple functions from practical time-keeping to emotional comfort in their accounts.

##### SC B1: Practical structure “day-to-day sound that you hear”

Sound functions as a memory aid and temporal marker (8 instances), supporting temporal structure through predictable patterns. Daily acoustic cues track routines: coffee makers mark mornings (SJT-04-P7, SJT-18-P6), laundry machines signal midday (SJT-07-P5), people returning from work indicate evening (MSJ-34-P5), cooking sounds signal mealtimes (SJT-05-P3), and wood burning marks evening transitions during the winter (MSJ-16-P6). Sound cues prompt planning and execution of activities: cooking sounds trigger meal preparation (“I can only hear the sounds of the steam cap! Just the idea that I’m going to purée the soup” MSJ-13-P3) while machine sounds facilitate time management (“We’re back, the washing machine’s on, I don’t have to do anything else, just load everything back into the closet in two hours, it’s so easy” MSJ-30-P5). These examples suggest that task engagement with domestic sounds serves as prompts for task planning and time management.

##### SC B2: Ritual and comfort “comforting process”

Regular acoustic markers establish domestic order and psychological comfort through familiarity. Daily sounds scaffold routines into meaningful rituals supporting practical and existential aspects of home life. Morning coffee-making creates a “comforting process” (SJT-18-P6) that structures each day’s beginning. Cooking sounds serve dual purposes—facilitating planning while creating emotional resonance. Evening transitions highlight this dual role, where wood burning creates temporal awareness and emotional security: “a home-like feeling of peace after a day’s work” (MSJ-16-P6). Familiar sounds connect to expressions of gratitude regarding age-related independence: “In the afternoon, the washing machine opens to dry, and the morning is over again, but it’s nice to have my machine working for me” (SJT-07-P5). This shows how soundscape listening tasks triggered both immediate task management and deeper reflection on daily patterns.

##### SC B3: Spatiotemporal integration “evening takes over quietly”

Everyday sounds function as temporal markers in domestic environments. Participants described how specific sounds distinguish different phases of the day and transitions between activities: “the ticking of our clock, the occasional rolling of wheels across the street of a car, the motor pulling up… People come home from their day’s work, the evening takes over quietly” (MSJ-34-P5). Another noted how “the tapping of wood burning and the soft buzzing of the fridge” creates a “home-like feeling of peace after a day’s work” (MSJ-16-P6). These examples show how soundscape structures temporal experience through distinct daily phases throughout the home. Drawing from patterns in household duties and routines described earlier (SC B1 & B2), sounds serve multiple spatiotemporal functions. Adapting prior soundscape research^52,53^, sounds support sense-making of space and time via auditory experience, with acoustic cues integrating temporal awareness with spatial understanding.

#### Theme C. Sound and memory associations

Reports illustrate how sounds helped create and maintain place attachment through memory associations. As introduced in the “Introduction” section, SoP refers to the psychological connection between people and their physical surroundings, specifically how people form emotional bonds with spaces through sensory experiences and memories. In this context, it explores how sounds create connections between people and their homes, shaping how they perceive their indoor soundscape.

##### SC C1: Sonic place-making “peace, warmth, and home-like comfort”

Soundscapes evoked emotional responses tied to specific places. The sound of a fire in a stove brought feelings of peace, warmth, and home-like comfort (MSJ-26-P5, SJT-18-P6); note that the study took place in winter. The stove’s fire represents more than thermal warmth—it signifies psychological comfort and spatial identity of the home. In participants’ accounts, sounds appeared to act as environmental anchors, connecting physical spaces with meaningful places through association patterns. In the context of sound and place experiences (“Introduction”), **association** refers to the process by which sounds trigger specific memories, emotions, or meanings, transforming spaces into meaningful places^28,29^. This connects to Raymond’s integration of affordance theory with SoP^22^, where environmental features create meaning through both immediate perception and longer-term processes.

##### SC C2: Memory bridge “brings me back”

Sound functions like a bridge between the present and the past, transporting individuals to significant life events with their associated emotions. Sounds evoked powerful nostalgic responses—falling rain triggered childhood memories: “bubbles falling on the ground, splashing open, a drop hanging from the wire, ten years ago we had a pond in our garden” (SJT-27-P5). Music served as a deliberate tool for reconnecting with past experiences and family memories (SJT-02-P3). Not all connections evoke positive reminiscence: “Industrial noises, buzzing noises… brings me back to 10 years ago, when we were lied to by our mayor, who, together with a neighbouring municipality, had 5.6 km^2^ evaluated to put up mega-reserves” (MSJ-31-P6). These examples show how sounds support autobiographical continuity by connecting past experiences to present moments. Note that other aspects of the EMA protocol specifically prompted for sound-memory connections, potentially influencing the reporting of such features in MSJ and SJT tasks.

#### Theme D. Social and cultural dimensions

Participants’ soundscapes operated across social scales, connecting intimate household interactions to broader cultural and environmental consciousness.

##### SC D1: Monitoring others “some chatter to hear at a distance”

Sound functions as a medium for detecting social presence in domestic spaces through human indicators like voices (MSJ-38-P2), footsteps (SJT-26-P7, MSJ-38-P2), and activity cues such as “what I noticed was [PARTNER] walking around me, and the sound of the water he was spraying on our plants” (SJT-07-P5). This extends to pets: “the dog was snoring, the cat was meowing” (MSJ-33-P1). Sonic cues allow tracking of family activities without direct interaction, similar to findings in ref. ^33^. This monitoring also applies to neighbourhood awareness, with participants noting sounds indicating patterns like “the neighbours are back from work” (MSJ-34-P5).

##### SC D2: Cultural connections “geese high in the air”

Sounds connect individuals to broader cultural and environmental contexts. This sensitising concept demonstrates how soundscapes bridge personal perception, civic awareness, and environmental consciousness. For example, specific sounds carry cultural significance and convey abstract concepts like how an airplane sound evokes feelings of freedom: “travel, freedom, the future” (MSJ-07-P6). These symbolic associations (e.g., airplanes representing possibility and mobility) create meaning through a symbolic mechanism of cognition, similar to how sounds in SC C1 symbolise warmth and home. Natural sounds prompted broader reflections about environmental concerns. Geese migration triggered ecological awareness: “probably a couple of geese high in the air, and those guys are getting ready for their overwintering” (SJT-31-P4). Similarly, rain sounds prompted reflections on flooding concerns in a neighbouring region (SJT-27-P5), while industrial noises connected to local governance controversies (MSJ-31-P6).

### Theoretical reflection on themes

We now position our findings in relation to established theory, demonstrating how these themes align with current understanding of indoor soundscape perception, situational factors, and sense of place for older adults.

Theme A (“Thematic analysis of sound experiences in the home” section) aligns with established research on indoor soundscape preference factors. Our findings align with previous work^19^ showing that older adults’ soundscape preferences are shaped by their perceived control over sound sources. Similarly, the importance of active control over sound is consistent with research showing that increased agency (“perceived noise control”^5^) mediates between noise exposure and noise annoyance^5^. The patterns in Theme A appeared across participants with varied living circumstances. For instance, expressions of frustration with uncontrollable sounds (SC A1) were reported by participants living both independently (P6, living alone with caregiving responsibilities) and with partners (P4 & P5, suburban couple). While we cannot generalise from this small sample, concerns about acoustic control appeared across different living arrangements and ages in our data, consistent with the interpretation that such concerns may relate to broader experiences of domestic soundscape agency.

The analysis of Theme B (“Thematic analysis of sound experiences in the home” section) sets out connections between spatiotemporal integration and contextual understanding in soundscape experiences. Recent work in soundscape research has emphasised the importance of considering non-acoustic contextual factors^18^. Our Theme B findings demonstrate how sounds function as spatiotemporal anchors in domestic environments, structuring daily routines and creating meaningful transitions between activities. These acoustic anchors align with the need for further understanding of the situational category of soundscapes^18^, which includes time-of-day and activity-related contextual elements that modify sound perception and experience.

Theme C (“Thematic analysis of sound experiences in the home” section) examined how soundscapes contribute to a sense of place. The theme illustrates how sounds evoke in-the-moment associations and reminiscence that strengthen place attachment to the concept of home. This also aligns with van den Bosch’s notion of ‘audible safety’^54^, wherein familiar soundscapes foster comfort through their predictability and consistency with expectations. The sound associations documented in SC C1 illustrate how familiar sounds serve as cognitive shortcuts to safety assessments, what van den Bosch’s framework would categorise as “pleasantly appraised environments” that “co-occur with pleasant inner affective states”^54^. These Theme C patterns align with pandemic-era findings that ideal domestic soundscapes (for work) combine quietness with positive, familiar sounds, supporting concentration, relaxation, intimacy and control^26^. Those case studies link familiarity and controllability of indoor sounds with comfort for relaxation and psychological well-being, underscoring how predictable sounds support attachment and everyday functioning^25,26^. These connections highlight how sound and memory interact with implications for older adults’ well-being, namely how soundscape design interventions could impact the predictability of sound experience in the home.

Our analysis of Theme D (“Thematic analysis of sound experiences in the home” section) demonstrates how soundscapes operate simultaneously at individual and collective levels. Beyond immediate social presence, participants connected everyday sounds to broader cultural and environmental awareness, suggesting soundscape listening offers an affordance for connecting people to wider environmental and political thoughts. This could align with research on soundscape and sustainability^55,56^, where soundscape listening has been linked to fostering behaviours that protect planetary health^57^.

### Listening experiences

Our analysis of MSJ and SJT data from the eight participants suggested several patterns in how they engaged with listening activities:

- Listening to Details: Several participants reported heightened attention to sound details, making them more present in the moment through activities like making coffee (SJT-04-P7), doing laundry (SJT-10-P4), and hearing outdoor noises (SJT-13-P3).
- Acceptance: Some participants demonstrated acceptance of potentially annoying sounds, recognising their necessity, as with the statement “as long as it’s temporary or intermittent, no problem” (MSJ-03-P6).
- Taking a Moment: For some, the tasks created space for reflection, with participants noting how “Noticing sounds and reflecting on them literally makes me stop for a moment” (SJT-04-P7).
- Noticing Regular Things: Participants reported new perspectives on mundane sounds, such as finding meaning in the “daily recurring sound” of a steam cooker (SJT-05-P3).
- Imaginative Listening: A few participants engaged with sounds poetically, describing rain as having “bright and clear” sounds, with “bubbles falling on the ground, splashing open” (SJT-27-P5).

These themes highlight how structured and repeated listening activities can cultivate everyday sonic experiences into opportunities for deeper engagement and personal insight.

### Participant survey feedback

Participants reflected on the study process via the *Post HDP Survey* (Stage 3) and interview follow-up. Survey questions shown in Fig. 2. Quotes indexed by participant ID and question response source, e.g., (P1:Q3). P8 did not answer any of the feedback questions via the online form, only well-being monitoring items, as we conducted a post-HDP interview to gather their answers to feedback questions. Analysis generated six themes describing participants’ experience.

First, participants reported **Enhanced Sound Awareness**, with improved environmental sound perception (P2, P4, P7), shifts from negative to positive sound focus (P2, P8), and greater appreciation for silence and nature (P2, P5, P8). Many characterised their experience as “fun” (P2:Q1, P5:Q1) and discovered their environment through “a new angle” (P2:Q1), noting how “ordinary sounds were suddenly fun” (P5:Q3). Second, many participants reported **Well-being and Growth** benefits, including positive effects from heightened sound awareness (P2, P4, P5), opportunities for self-discovery (P5, P6, P8), and distraction from physical pain (P5). However, two participants suggested that including more group activities would enhance the experience (P6, P8).

Participants noted **Technical Challenges**, including navigation issues with the app interface (P1, P2, P4), needs for simpler instructions (P4, P5), and recording functionality problems (P2, P7). Some criticised the app interface complexity and volume of materials (P4:Q1, P4:Q7, P2:Q7), with one participant reporting stress from notification timing (P4:Q3). The **Recording Device Impact** varied, with some experiencing initial discomfort (P2, P3, P6), behavioural changes during conversations (P6, P8), while others had minimal device awareness (P1, P4).

Participants gained **Enjoyment** from the study, including social elements like workshops (P3, P8), valuable researcher and panel manager interactions (P3, P5). Some found the experience “challenging” (P2:Q3, P6:Q1), “inspiring” (P6:Q1), and informative (P3:Q1). Finally, feedback on **Study Structure** noted intensity after several days (P3), suggestion that more thoughtful responses developed over time (P8), and needs for timing improvements (P4, P7).

## Discussion

SEAM, as a hybrid of EMA and Cultural Probe methods, combines soundscape assessment with deep listening practices. In this study, it enabled us to explore the nuanced and contextual nature of our participants’ indoor soundscapes. The thematic analysis both maps established dimensions (“Theoretical reflection on themes” section) while revealing how sounds interact with daily routines, emotional states, and place attachment for the participants. This combined methodological and theoretical contribution generates rich, contextually relevant data about the residential settings we were designing for.

The participant experiences documented in sections “Listening experiences” and “Participant survey feedback” suggest SEAM’s potential as both a research tool and an intervention. Enhanced sound awareness (“Participant survey feedback” section) indicates the protocol supported deeper engagement with everyday soundscapes, allowing participants to access and articulate experiences that might otherwise remain unnoticed. The protocol offered structured opportunities for immediate perception (e.g., *Taking a Moment*, “Listening experiences” section) and reflective engagement (e.g., *Imaginative Listening*, “Listening experiences” section), with complementary physical probes and digital EMA tasks enabling multiple modalities of documentation. Beyond data collection, the structured engagement with everyday sounds may itself constitute the basis for *sonic well-being* interventions, potentially offering cognitive stimulation through heightened sensory attention and memory activation for older adults aging-in-place. Such an application would require adapting the protocol specifically for intervention delivery.

The themes and positioning developed from SEAM capture aspects of *how* our eight older adults construct meaning from domestic soundscapes: through personal agency in shaping acoustic environments, temporal routines structured by sound, sound-memory associations fostering place attachment, and social presence detected through acoustic monitoring. These sense-making processes operated across participants’ varied living circumstances—from urban flats to rural homes, across a 20-year age span, and in both solo and partnered households. The purpose was to gather requirements for designing supportive indoor soundscapes; the themes offer transferable design insights rather than universal claims. Different populations may attach meaning to different sound sources or hold different preferences, but interventions that support personal acoustic control, temporal anchoring, memory associations, and social connection could address design-relevant needs beyond our specific context. Exploring how these processes manifest in other populations and domestic care settings remains important future work.

Linking back to Theme A on agency and environmental response (“Thematic analysis of sound experiences in the home” section), recent work on Acoustic Personalised Environmental Control Systems (Acoustic PECS)^58^ demonstrates that systems providing individually controlled acoustic environments in occupants’ immediate surroundings can reduce annoyance, improve concentration, and enhance perceived control in challenging acoustic environments. This framework suggests a direction for Audio AI research: systems that enable occupants to personalise their soundscapes through adaptive masking, selective sound enhancement, or localised sound zoning could address the control-comfort relationship identified in Theme A while respecting the diverse preferences revealed in our participant data.

Addressing practical issues with the SEAM methodology, we identified several areas for protocol refinement. Technical challenges with the app interface created frustration for some participants (“Participant survey feedback” section), while the notification schedule caused stress for others (“Participant survey feedback” section). Some participants found the protocol intensity challenging after several days (“Participant survey feedback” section), and data imbalance occurred with certain participants contributing disproportionately (“Data collection overview” section). Moving forward, we recommend the following protocol improvements for future EMA studies investigating older adult soundscapes. These refinements aim to enhance participant experience and data richness while maintaining the interpretive depth that characterises the SEAM approach:

- **Personalised scheduling**: Allow participants to set their preferred number and timing of daily EMA notifications.
- **Simplified protocol design**: Reduce task variety and focus specifically on SJT and MSJ responses to decrease participant burden.
- **Enhanced contextual data**: Gather specific room location data within homes to better understand spatial dimensions of soundscape perception and place attachment.

Borrowing from broader soundscape research^41^, we additionally recommend implementing *multimodal cues* to capture embodied responses to sounds and establishing *iterative feedback loops* where participants can review their own categorisations, which can provide deeper insights into perceptual and associative processes.

### Limitations

This study’s interpretive insights should be understood within specific methodological and contextual boundaries. The eight participants were recruited from Flemish Belgium, all had stable housing arrangements, and demonstrated willingness to engage reflectively with domestic soundscapes. This sample enabled exploration of varied domestic circumstances (urban flats to rural homes with caregiving responsibilities, ages 56–76) but does not represent the oldest-old populations (80+), institutional care settings, or households facing housing precarity. The SEAM protocol privileges articulacy and digital literacy; participants who find verbal reflection challenging or lack smartphone access would engage differently with the method. Engagement varied considerably, with some participants completing all requested tasks and others submitting minimal responses, reflecting both individual preference and the burden of combined EMA and Cultural Probe activities over a week-long deployment. The broader study context–including workshops, home audio recording, and regular contact with researchers and the Living Lab panel manager–likely shaped participants’ openness to soundscape reflection and is likely to have influenced the positive feedback reported in the “Participant survey feedback” section. Future applications of SEAM in other cultural contexts, care settings, or with populations facing cognitive or sensory impairments would provide valuable opportunities to refine the protocol for different lived experiences of home and to explore how the identified sonic sense-making processes manifest across diverse circumstances.

In this paper, we have introduced the Soundscape Experience Activities and Mapping (SEAM) method for understanding older adults’ indoor soundscape experiences. SEAM provided a lens to examine how sounds function as spatiotemporal anchors, structure daily routines, and foster place attachment through memory. The complementary nature of our soundscape tasks (Momentary Soundscape Judgements, Sound Journaling Tasks, Cultural Probe) enabled both immediate soundscape assessment and deeper reflection. This work demonstrates how situated methods can access experiential dimensions of home soundscapes relevant to designing supportive environments for ageing-in-place.

## Supplementary information

Supplementary Information

## Data Availability

The complete bilingual protocol—including all listening tasks, prompts, and response formats—is available in our workflow supplement (https://zenodo.org/records/14946672). All anonymised data is available via the data supplements (https://zenodo.org/records/14999348 and ref. ^59^). The customised Avicenna Research app is not available at the time of submission, but the bilingual JSON setup files for each task will be added to the V2 of the workflow supplement on Zenodo.
