# Supplementary material for: Soundscape experience activities and mapping
Source: NPJ Acoust. 2026 Feb 23;2(1):7. doi: 10.1038/s44384-025-00041-6 (PMC12929050; doi:10.1038/s44384-025-00041-6)
Supplement: Supplementary file 1 — Supplementary Information [file 44384_2025_41_MOESM1_ESM.pdf]

Soundscape Experience Activities and Mapping: Supplemental Information

Thomas Deacon[1*], David Frohlich[2] and Mark D. Plumbley[1]

[1*]Centre for Vision, Speech and Signal Processing, University of Surrey, Stag Hill Campus, Guildford, GU2 7XH, Surrey, UK.
[2]Digital World Research Centre, School of Arts, Humanities and Creative Industries, University of Surrey, Stag Hill Campus, Guildford, GU2 7XH, Surrey, UK.

*Corresponding author(s). E-mail(s): t.deacon@surrey.ac.uk;
Contributing authors: d.frohlich@surrey.ac.uk; m.plumbley@surrey.ac.uk;

Abstract

This paper presents Soundscape Experience Activities and Mapping (SEAM), a new method for exploring how older adults perceive and relate to their indoor acoustic environments. With global ageing, populations are increasingly choosing to age in place, creating opportunities to enhance older life through the intentional design of supportive home soundscapes. Through a mixed-method approach combining Ecological Momentary Assessment with Cultural Probe methods, we engaged eight older adults (age 56–76) in Belgium to document their domestic soundscape experiences. Reflexive thematic analysis constructed four patterns of meaning: personal agency in shaping acoustic environments, temporal routines structured by sound, sound-memory associations fostering place attachment, and social presence through acoustic monitoring. Within this study context, sounds functioned as spatiotemporal anchors, structuring daily routines while fostering place attachment through memory. This exploratory design research offers situated insights for soundscape interventions that support independence, while highlighting methodological considerations for situated soundscape research.

Keywords: indoor soundscapes, older adults, ecological momentary assessment, sense of place, thematic analysis

Supplemental Information

Abbreviations

- AI: Artificial Intelligence
- AI4S: AI for Sound project
- EGA: Ethics Governance Approval
- EMA: Ecological Momentary Assessment
- EPSRC: Engineering and Physical Sciences Research Council
- GDPR: General Data Protection Regulation
- HCI: Human–Computer Interaction
- HDP: Home Deployment Phase
- ISO: International Organization for Standardization
- JSON: JavaScript Object Notation
- KU Leuven: Katholieke Universiteit Leuven
- LiCalab: Living and Care Lab (Belgium)
- MSJ: Momentary Soundscape Judgements
- PID: Participant ID
- RIGO: Research Integrity and Governance Office (University of Surrey)
- RTA: Reflexive Thematic Analysis
- SC: Sensitising Concept
- SEAM: Soundscape Experience Activities and Mapping
- SMEC: Social and Societal Ethics Committee (KU Leuven)
- SJT: Sound Journaling Tasks
- SoP: Sense of Place
- VITALISE: Virtual Health and Wellbeing Living Lab Infrastructure (EU project)
- VAS: Visual Analogue Scale
- YIR: Years in Residence

Supplementary Note 1

Data Collection Details

Table S1 shows data collection results and compliance rates across 8 participants (P1-P8) for the MSJ (Momentary Soundscape Judgments) and SJT (Sound Journaling Tasks) data. Compliance rates were calculated based on the number of prompted submission requests during the study period (3 for SJT and 7 for MSJ). Only valid entries with usable data for thematic analysis are counted, compliance rate capped at 100% to ameliorate over participation skewing average e.g. P3 SJT responses. SJT responses showed high compliance rates (Avg. 75%, σ: 30%) with responses ranging from 1 to 20 per participant, totalling 38 with a median of 3. MSJ responses had similar compliance (Avg. 64%, σ: 33%), ranging from 1 to 9 responses per participant, totalling 38 with a median of 5. Participant P3 showed very high SJT engagement with 20 entries, while P6 over responded on the MSJ task with 9 entries.

Participants demonstrated moderate overall engagement with compliance rates capped at 100% to avoid over-participation skewing the metric. While some participants achieved full compliance, others showed minimal engagement. This engagement

pattern is consistent with other EMA studies where participation tends to vary significantly across participants. For example, in wellbeing-focused EMA studies, participants completed on average 72% (σ= 14%) of all EMAs with a range of 43–95% [1]. Our average compliance rate of 68% (σ: 31%) suggests our data matches average engagement for EMA studies, though with greater variability across participants.

Notable patterns include exceptional contributions from P3, who submitted 20 SJT entries well above the requested 3, and P6, who completed 9 MSJ entries compared to the requested 7. This over-participation from some participants demonstrates high engagement with the protocol but required capping compliance rates at 100% to avoid skewing average metrics.

Supplementary Table S1 Participant response rates and compliance for Sound Journaling Tasks (SJT) and Momentary Soundscape Judgments (MSJ) data collection. σ = standard deviation

**Participant**	**SJT Responses (Compliance %)**	**MSJ Responses (Compliance %)**
P1	2 (67%)	3 (43%)
P2	1 (33%)	1 (14%)
P3	20 (100%)	6 (86%)
P4	3 (100%)	6 (86%)
P5	4 (100%)	7 (100%)
P6	5 (100%)	9 (100%)
P7	2 (67%)	2 (29%)
P8	1 (33%)	4 (57%)
**Total**	38	38
**Median**	3	5
**Requested Submissions**	3	7
**Avg. Compliance (σ)**	75% (30%)	64% (33%)
**Weighted Avg. Compliance (σ)**	68% (31%)	

Examining compliance rates across the 8 participants we can see some participants achieved full compliance (notably P3, P4, P5, and P6 with 100% SJT compliance), while others showed minimal engagement (P2 and P8 with 33% SJT compliance, submitting just 1 response each). The median responses (3 SJT and 5 MSJ) and average compliance rates (75% for SJT, 64% for MSJ) indicate moderate overall engagement.

Acknowledgements. The authors would like to thanks all the research and administration staff at LiCalab who assisted in the research process and execution, specific thanks to Kim Helsen (Researcher), Leen Broeckx (Panel Manager), and Vicky Van der Auwera (Operations Manager).

Funding. This work was supported by Engineering and Physical Sciences Research Council (EPSRC) Grant EP/T019751/1 "AI for Sound (AI4S)". The Transnational Access grant that supported work with LiCalab was funded by the European Commission, Grant agreement ID: 101007990 "VITALISE".

Data and Materials availability. The complete bilingual protocol – including all listening tasks, prompts, and response formats – is available in our workflow supplement (https://zenodo.org/records/14946672). All anonymised data is available via the data supplements (https://zenodo.org/records/14999348 and [2]). The customised Avicenna Research app is not available at time of submission, but the bilingual JSON setup files for each task will be added to the V2 of the workflow supplement on Zenodo.

Ethics approval and consent to participate. Participants physically signed informed consent for the study and data analysis with support from the LiCalab panel manager, who explained the protocol and addressed queries during the Stage 1 kick-off workshop. LiCalab maintained paper records to preserve participant anonymity, with authors using only numerical participant IDs throughout the data collection and documentation process. The study received ethical approval from all required boards (University of Surrey RIGO: FEPS 23-24 001 EGA and KU Leuven SMEC: G-2023 11 2174). All informed consent statements and information available in workflow supplement (https://zenodo.org/records/14946672).

References

[1] de Vries, L. P., Baselmans, B. M. L. & Bartels, M. Smartphone-based ecological momentary assessment of well-being: A systematic review and recommendations for future studies. *Journal of Happiness Studies* **22**, 2361–2408 (2021).

[2] Bibbó, G., Deacon, T., Singh, A. & Plumbley, M. D. *The Sounds of Home: A Speech-Removed Residential Audio Dataset for Sound Event Detection*, 49–53 (2024).
